# Conserved Expression of the Glutamate NMDA Receptor 1 Subunit Splice Variants during the Development of the Siberian Hamster Suprachiasmatic Nucleus

**DOI:** 10.1371/journal.pone.0037496

**Published:** 2012-05-31

**Authors:** Giles E. Duffield, Jens D. Mikkelsen, Francis J. P. Ebling

**Affiliations:** 1 Department of Biological Sciences, University of Notre Dame, Notre Dame, Indiana, United States of America; 2 Neurobiology Research Unit, Rigshospitalet, Copenhagen University Hospital, Copenhagen, Denmark; 3 School of Biomedical Sciences, University of Nottingham Medical School, Nottingham, United Kingdom; Pennsylvania State University, United States of America

## Abstract

Glutamate neurotransmission and the N-methyl-D-aspartate receptor (NMDAR) are central to photic signaling to the master circadian pacemaker located in the hypothalamic suprachiasmatic nucleus (SCN). NMDARs also play important roles in brain development including visual input circuits. The functional NMDAR is comprised of multiple subunits, but each requiring the NR1 subunit for normal activity. The NR1 can be alternatively spliced to produce isoforms that confer different functional properties on the NMDAR. The SCN undergoes extensive developmental changes during postnatal life, including synaptogenesis and acquisition of photic signaling. These changes are especially important in the highly photoperiodic Siberian hamster, in which development of sensitivity to photic cues within the SCN could impact early physiological programming. In this study we examined the expression of NR1 isoforms in the hamster at different developmental ages. Gene expression in the forebrain was quantified by in situ hybridization using oligonucleotide probes specific to alternatively spliced regions of the NR1 heteronuclear mRNA, including examination of anterior hypothalamus, piriform cortex, caudate-putamen, thalamus and hippocampus. Gene expression analysis within the SCN revealed the absence of the N1 cassette, the presence of the C2 cassette alone and the combined absence of C1 and C2 cassettes, indicating that the dominant splice variants are NR1-2a and NR1-4a. Whilst we observe changes at different developmental ages in levels of NR1 isoform probe hybridization in various forebrain structures, we find no significant changes within the SCN. This suggests that a switch in NR1 isoform does not underlie or is not produced by developmental changes within the hamster SCN. Consistency of the NR1 isoforms would ensure that the response of the SCN cells to photic signals remains stable throughout life, an important aspect of the function of the SCN as a responder to environmental changes in quality/quantity of light over the circadian day and annual cycle.

## Introduction

Circadian rhythms are an integral component of physiology and behavior, and the master circadian oscillator in the mammal is located within the suprachiasmatic nucleus (SCN) of the hypothalamus. The SCN clock is entrained to the light-dark (LD) cycle by photic signals from the retina via a monosynaptic retinohypothalamic tract (RHT) input to the SCN. Glutamate is the predominant neurotransmitter of the RHT and mediator of light entrainment cues in the adult mammal (e.g. [1] and reviewed in [2], [3]). Moreover, glutamate is involved in the regulation of developmental events in various brain regions, and in particular retino-recipient areas [4], [5]. The N-methyl-D-aspartate (NMDA) type of glutamate receptor is of particular interest since it has been demonstrated to play a central role in the transduction of photic cues in the SCN [3], [6], and critical to normal development of visual pathways and associated with synaptogenesis and synaptic plasticity [4], [5], [7], [8]. Thus, it is possible that glutamate may play a role in the innervation of the SCN by the RHT, in synaptogenesis of the SCN during the postnatal period and in the functional switch between maternal to photic derived cues for entrainment of the circadian clock [9]–[13]. Moreover, these events in the SCN might be associated with changes in glutamate receptor subtype expression, with either a switch in expression being causal to a developmental event, or conversely, the switch being driven by a developmental event. *In situ* hybridization and Reverse transcription polymerase chain reaction (RT-PCR) studies indicate that several glutamate receptor genes are expressed in the SCN [14]–[18]. Developmental changes in glutamate receptor subunit expression occur in many brain regions in the rodent; e.g. expression of NMDAR2B (NR2B) and NR2D declines in several regions postnatally whilst expression of NR2A and NR2C increases [19], [20], and in the rat SCN, metabotropic glutamate receptor 1 (mGluR1)-immunoreactivity is higher in the early neonate than in the adult [21].

The NMDA receptors (NMDARs) are glutamate-gated ion channels composed of heterodimeric subunits, and the NMDAR1 (NR1) sub-unit is present in all functional receptor complexes [22]. The gene for the common subunit of the NR1 can be alternatively spliced at three positions, giving rise to up to eight different receptor isoforms. The alternatively spliced exons encode a 21 amino acid sequence in the N-terminus domain (N1), and adjacent sequences of 37 and 38 amino acids in the C-terminus domain (C1 and C2). Splicing out the exon segment that encodes the C2 cassette removes the first stop codon, resulting in a new open reading frame that encodes an unrelated sequence of 22 amino acids (C2′) before a second stop codon is reached. Differential RNA splicing has been shown to alter the structural, physiological and pharmacological properties and thus functional properties of receptors that comprise NR1 subunits (e.g. [23], [24]). Developmental changes in splicing of the NR1 gene have also been described [25]–[27].

The aim of the current study was to investigate the developmental regulation of NR1 mRNA isoforms in the Siberian hamster SCN. The Siberian hamster (*Phodopus sungorus*) was studied because the early development and photic regulation of the circadian system is necessary for the generation of rhythms of melatonin secretion from the pineal gland. This pattern of melatonin secretion provides the key seasonal time cue regulating somatic and reproductive development in this species [28]. The measurement of seasonal photoperiod information begins before birth, *via* maternal melatonin secretion [29]. However, postnatally the young must acquire the ability to respond directly to the environmental LD cycle, so the ontogeny of photic regulation of the circadian system and thus of melatonin secretion is of functional importance for this species.

The postnatal period in the rodent shows extensive functional and anatomical reorganization within the SCN, including synaptogenesis and innervation by the RHT [30]–[32], loss of sensitivity to maternal entrainment signals and acquisition of light responsiveness. We have previously demonstrated in the Siberian hamster that the RHT begins to grow into the ventrolateral SCN on postnatal day (PD) 3, has an exuberant innervation of the SCN by PD6 and is subsequently pruned to generate the adult pattern [11]. Also a photic response (light-induced immediate early gene c-Fos expression) first occurs in the SCN on PD3 [10]. During the late prenatal and early postnatal period the rodent circadian clock is entrained via signals derived from the mother [9], [12], [13], [33]–[37]. The photic response develops gradually during the first 2 weeks of postnatal life to eventually override the maternal entrainment mechanism [10]–[12], [33], [37], [38]. This results in the loss of certain signals and neuronal pathways associated with maternal entrainment, such as a dopaminergic input to the SCN [11]–[13], [33]. Therefore, NR1 splice variant gene expression was studied at key stages in the development of the SCN on PD2 (pre-innervation of the SCN by the RHT), PD6 (post-innervation and post-initiation of photic responsiveness) and in the adult (post-synaptogenesis, post-pruning of RHT fibers and post-maternal entrainment signaling).

*In situ* hybridization gene expression analysis was carried out on coronal sections at these ages, using probes complementary to common and variable regions of the rat mRNA. Hybridization of the common probe occurred ubiquitously in the brain, including the SCN at PD2 and at all subsequent ages. A probe complementary to deletion II/exon 22, and one detecting the absence of both the exon 21 and exon 22 regions, were also abundant in the SCN at all ages. In contrast, no hybridization of probes complementary to insertion I/exon 5 or to deletion I/exon 21 were detected in the SCN at any age. These observations suggest that NR1 is present in the SCN early in development, prior to RHT innervation and synaptogenesis, and before the age at which light can induce cellular changes within the SCN. These data suggest that the predominant isoforms in the SCN at all postnatal ages are NR1_001_ (NR1-2a) and NR1_000_ (NR1-4a). The fact that the SCN showed a consistent expression of NR1_001_ and NR1_000_ at all ages examined suggests that the ontogeny of the postnatal hamster SCN is not associated with a switch in NR1 subclass or a change in its level its expression. This is different to other brain structures such as the thalamus and hippocampus where we do observe developmental changes in the levels of NR1 isoforms. Whilst the conserved nature of high expression does suggest that the NR1 subunit is important in the regulation of SCN function throughout postnatal development, we provide no evidence that postnatal development events are regulated by an alteration of NR1 isoform.

## Methods

### Animals

Siberian hamsters (*Phodopus sungorus*) were obtained from the breeding colony in the Department of Anatomy, University of Cambridge [10], [11], [33], and rats (*Rattus rattus*) were obtained from Harlan UK Ltd., Bicester, Oxford, UK, and maintained on a 16∶8 or 12∶12 light∶dark photoschedule, respectively (light, 300–400 lux; dark, <14 lux dim red light). The day of birth was assigned as postnatal day 1, PD1 [10], [39]. Tissue was harvested at Zeitgeber time (ZT) 7–12 (ZT12 being time of lights OFF). Siberian hamsters aged PD2 (n = 13), PD6 (n = 13) and adult (n = 10), and adult rats (n = 3) were killed by cervical dislocation and the brains rapidly removed, frozen on dry ice and stored in a freezer (−50°C) prior to cutting on a cryostat. All animal procedures were carried out under license according to the United Kingdom Home Office Animals (Scientific Procedures) Act of 1986 (Project license PPL 80/0474).

### *In situ* hybridization

**Figure 1:**
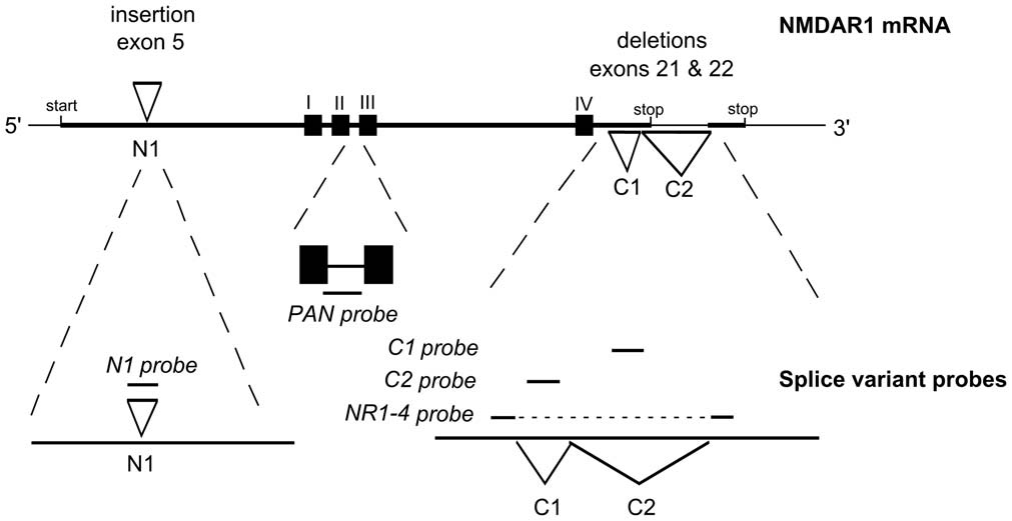
Schematic diagram of NR1 mRNA structure showing positions of the oligonucleotide probes. Note the position of three alternatively spliced exons in the mRNA (upper) and the complementary positions of the splice-specific probes and of a probe identifying a conserved region (lower). Of the mRNA, note the position of the coding areas for the transmembrane domains I to IV (filled squares); the 5′ insertion of exon 5, also known as N1; the 3′ deletions of exon 21 and 22, also known as C1 and C2, respectively; and the start codon and the two alternative stop codons. The positions of the oligonucleotide probes are represented by the short solid lines against a magnified region of the mRNA. Dotted lines reveal the area of the mRNA that has been magnified. Adapted from Laurie and Seeburg [25].

*In situ* hybridization (ISH) was carried out on 16 µm serial coronal sections, cut at the level of the SCN and caudate-putamen and as detailed previously [11], [33]. Slides were hybridized with ^35^S labeled oligonucleotide probes (36 mer–48 mer) complimentary to the rat NMDAR1 gene [25], [40], [41]. Five different probes were used to identify the following: a common region to all NR1 mRNA (PAN); the N1 cassette, insertion I/exon 5 (N1 probe); the C1 cassette, deletion I/exon 21 (C1 probe); the C2 cassette, deletion II/exon 22 (C2 probe); and the absence of both the C1 and C2 cassettes (NR1-4) (see Figure 1). Probe sequences are shown in Table S1. The N1 probe identifies isoforms NR1-4b, NR-2b, NR1-3b and NR1-1b, C1 probe identifies isoforms NR1-3a, NR1-1a, NR1-3b and NR1-1b, C2 probe identifies isoforms NR1-2a, NR1-1a, NR1-2b and NR1-1b, and NR1-4 probe indentifies isoforms NR1-4a and NR1-4b. See Table 1 for details of the insert components and different nomenclature. Slides were exposed to autoradiography film (Hyperfilm MP, Amersham, UK) for 7–21 days at 4°C. Slides containing sections from animals at different ages were processed together with the same probe, and these were also exposed together on the same film (see below). To reveal hybridization at the cellular level, slides processed with the PAN and C2 probes were also dipped in photographic emulsion (K5, Ilford, UK) and developed after 21–64 days, and counterstained with cresyl violet. Arginine Vasopressin (AVP) and Vasoactive intestinal polypeptide (VIP) probes complimentary to their respective mRNAs were used as markers of the SCN [42], [43] (Table S1). AVP and VIP identify the dorso-medial and ventro-lateral zones of the SCN respectively. These probes, and antibodies identifying their protein products, have been used routinely for the purpose of defining the SCN region in the rodent on alternate sections of the same brain [3], [10], [11], [39]. For alternate sections used for NR1-4 analysis, and as a counter stain in emulsion dipped PAN and C2 labeled sections, cresyl violet Nissl staining was used to aid in determination of SCN location: SCN was revealed as a darker region of the hypothalamus by virtue of its high neuron density. Three ISH control procedures were carried out on sections processed alongside sections hybridized with the labeled probe and exposed on the same photographic film: hybridization with labeled probe combined with 100 times excess unlabelled probe; treatment with RNAse A prior to hybridization with the labeled probe; and in the case of NR1 (C2), hybridization with labeled C2 sense probe. Adult rat brains were included in the study to control for any obvious forebrain regional differences in hybridization for any of the probes used between the rat and Siberian hamster.

**Table 1:** Insert components and commonly used nomenclature for the eight NMDAR1 splice variants.

Splice variant (a)	Insert			Other names with References			
	N1	C1	C2	(b)	(c)	(d)	
NR1_000_	−	−	−	NR1-4a	R1E		
NR1_001_	−	−	_+_	NR1-2a	R1C	NMDA-R1C	
NR1_010_	−	_+_	−	NR1-3a	R1D		
NR1_011_	−	+	+	NR1-1a	R1A	NMDA-R1A	
NR1_100_	+	−	−	NR1-4b	R1G		
NR1_101_	+	−	+	NR1-2b	R1F		
NR1_110_	+	+	−	NR1-3b			
NR1_111_	+	+	+	NR1-1b	R1B	NMDA-R1B	

The subscript (1) of NR1 indicates the presence, and (0) the absence, in left to right positions, corresponding to the three alternatively spliced axons in the 5′ to 3′ direction, N1, C1 and C2. The N1, C1 and C2 probes detect the presence of each of the three cassettes, and the NR1-4 probe detects the absence of both C1 and C2 cassettes; (a) Durand *et al.* [23]; (b) Hollmann *et al.* [80]; (c) Sugihara *et al.* [81]; and (d) Nakanishi *et al.* [82]; Table adapted from Zukin and Bennett [54].

Autoradiographic films were scanned with a Hamamatsu CCD camera (Hamamatsu k.k., Hamamatsun-city, Japan) and analyzed using Image J software. For each section the mean pixel density (measured as a grey-scale value from 1 to 256) was determined for each specific forebrain region in 2–4 sections per animal. Values were background corrected against the mean pixel density value for the corpus callosum. The corpus callosum was chosen as the control area because it was assumed that it contains little NMDA receptor mRNA [44]. *Relative grey scale values* were calculated by dividing the specific region measure by the corpus callosum measure taken from the same section. Emulsion-dipped sections were analyzed by a silver grain counting procedure using Image J. Sections were stained by cresyl violet to define brain structures of interest. Grains were counted over cells within a 120 µm^2^ area of brain section in the regions of the SCN and piriform cortex. Grains were also counted in the corpus callosum, but throughout the 120 µm^2^ area and not specifically over the few cells present. Mean values were determined for each animal from at least 3 sections, and at least 3 animals were used to derive a group mean.

### Immunohistochemistry

Immunohistochemical analysis (IHC) of NR1-immunoreactivity was performed as previously described [10] using an anti-NR1 rabbit polyclonal antibody (Chemicon, Temecula, CA, USA) at a dilution of 1∶100. Its production, characterization and validation for IHC has been described previously for the rat [45] and has been used previously in studies of the Siberian hamster [46]. The antiserum recognizes a C-terminal epitope of NR1, and would therefore be expected to detect the NR1-1a, 1b, 2a, and 2b splice variants, but not NR1-3a, 3b, 4a or 4b splice variants.

### Statistical analysis

Data were analyzed by one-factor ANOVA to determine age-related changes in NR1 splice variant expression, followed by Dunnett's *post-hoc* t-tests. Significance was set at the P<0.05 level.

## Results

Regional binding of the probes within the brain of both rat and Siberian hamster was conserved and consistent with the observations of Standaert *et al.* [41] and Laurie and Seeburg [25]. To be confident in the specificity of the probes in the Siberian hamster brain, the current study first repeated this study in the adult rat and hamster and then concentrated on a series of brains from different aged hamsters.

**Figure 2:**
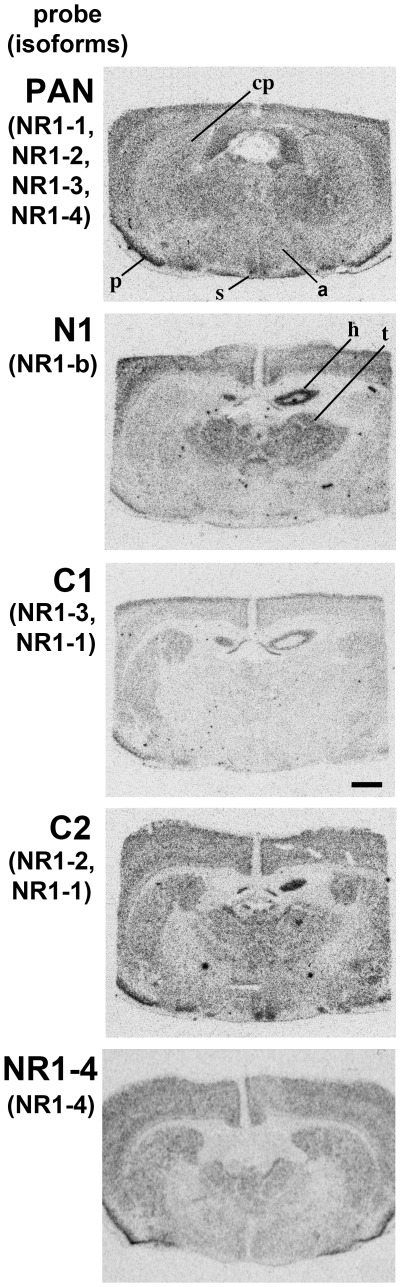
Hybridization of NR1 variable region probes in Siberian hamster forebrain. Shown are the PAN (top), N1, C1, C2 and NR1-4 (bottom) probes in the adult Siberian hamster brain. Autoradiograph of alternate coronal sections taken through the forebrain at the level of the SCN from a representative animal. The sections representing the PAN probe and the NR1-4 hybridization are taken from different animals (top and bottom). a, anterior hypothalamus; cp, caudate putamen; h, hippocampus; p, piriform cortex; s, suprachiasmatic nucleus; t, thalamus. Scale bar = 1000 µm.

Figure 2 and Figure S1 show representative hybridized sections from the adult hamster and adult rat, respectively, and Table 2 and Table 3 show a general assessment from autoradiographs of regional binding of NR1 probes in adult Siberian hamster and adult rat forebrain, respectively. The splice variant probes showed discriminate hybridization to various brain regions. For example, the N1 probe hybridized weakly in the caudate-putamen, whilst the C1 probe hybridized weakly in both caudate-putamen and thalamus (Table 2 and Table 3). Only the C2 probe hybridized strongly in the hypothalamus, and in particular the SCN (Table 2 and Table 3), and are similar to the observations made by Laurie and Seeburg [25]. Note that the patterns of hybridization for all the probes were comparable between the two species.

**Table 2:** Hybridization of NR1 oligonucleotide probes in adult Siberian hamster brain.

	Brain Region					
Probe	SCN	anterior hypo-thalamus	piriform cortex	caudate-putamen	thalamus	hippo-campus
PAN	+++	++	++++	++	++	++++
N1	0	0	+++	+	+++	++++
C1	0	0	+++	+	0	+++
C2	+++	+	++++	++	++	++++
NR1-4	+	+	+++	+	+	++

Summary of regional expression of NR1 splice variants in the adult Siberian hamster brain, based on film autoradiograph images. Relative abundance is presented as an arbitrary within-species relative scale, indicated as none (0), low (+), moderate (++), high (+++), or very high (++++).

**Table 3:** Hybridization of NR1 oligonucleotide probes in adult rat brain.

	Brain Region					
Probe	SCN	anterior hypo-thalamus	piriform cortex	caudate- putamen	thalamus	hippo-campus
PAN	+++	++	++++	++	++	++++
N1	0	0	+	+	+++	+++
C1	0	0	++	+	0	++
C2	+++	++	++++	+++	+++	++++

Summary of regional expression of NR1 splice variants in the adult rat brain, based on film autoradiograph images. Relative abundance is presented as an arbitrary within-species relative scale, indicated as none (0), low (+), moderate (++), high (+++), or very high (++++).

**Figure 3:**
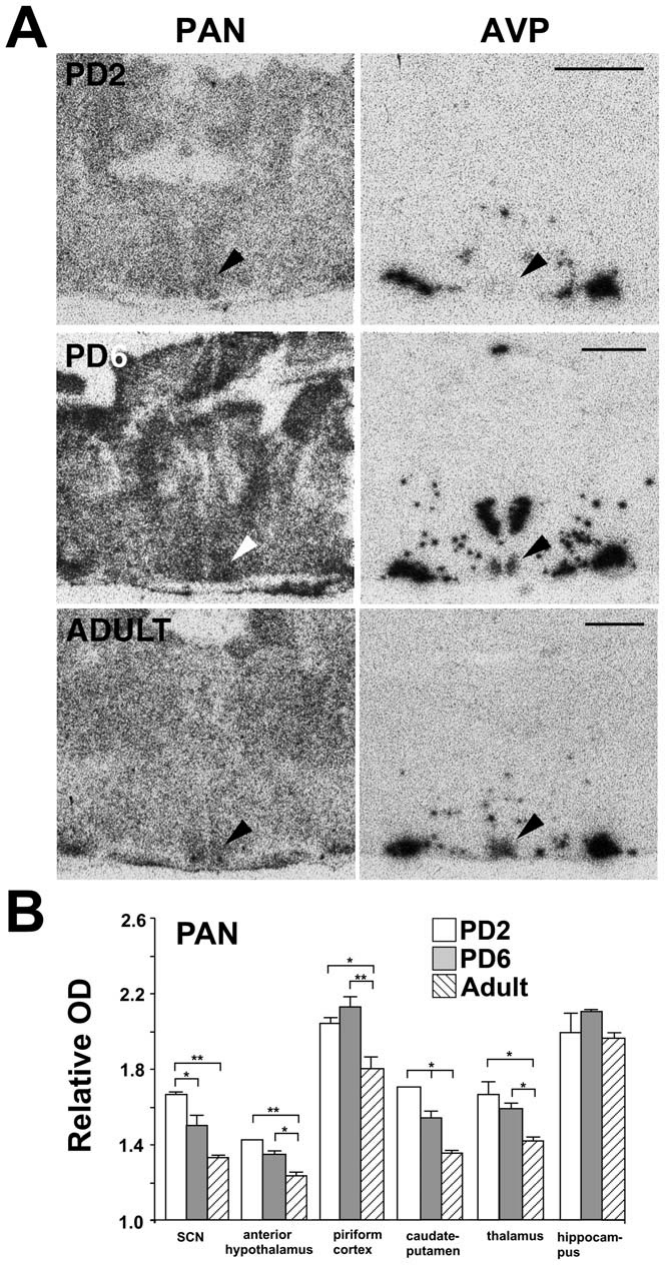
NR1 gene expression in the Siberian hamster forebrain at different developmental ages. (A) Hybridization of the NR1 conserved region probe, PAN (left), and arginine vasopressin (AVP) (right). Autoradiographs of coronal sections taken through the Siberian hamster forebrain at the level of the SCN (arrow) at the ages PD2 (top), PD6 and adult (bottom). AVP identifies the dorso-medial zone of the SCN. Note low level of AVP expression at PD2, in line with published reports in the rat during early development [79]. The PD2 section presented for the PAN probe is of a lower exposure time, allowing for better resolution of SCN region. Quantification was conducted on sections receiving equal exposure time. Scale bars = 1000 µm. (B) Quantification of PAN probe hybridization in various forebrain regions in hamsters of ages PD2, PD6 and adult. Brain regions are suprachiasmatic nucleus (SCN), anterior hypothalamus, piriform cortex, caudate-putamen, thalamus, and hippocampus. Probe hybridization signal was measured as relative optical density (OD), representing relative levels of gene expression (OD of specific brain region divided by OD of corpus callosum in same section). Values represent the mean ± standard error of the mean (SEM) relative OD values from 3 to 5 animals. *P<0.05 and **P<0.01 when regional relative OD is age compared (Dunnett's *post-hoc* t-test).

The PAN probe hybridized ubiquitously in the hamster and rat forebrain (Figure 2 and Figure S1), and in the hamster this pattern did not change with age. In the SCN, binding was distinct and more prominent than the surrounding anterior hypothalamus at all ages (Figure 3A). Figure 3B summarizes the analysis of the effect of age on the levels of relative hybridization of the PAN probe in forebrain regions in the Siberian hamster. One-factor ANOVA revealed a significant reduction in relative hybridization of PAN with age in the SCN (F_2,10_ = 8.2, P<0.01), anterior hypothalamus (F_2,10_ = 7.3, P<0.05), piriform cortex (F_2,11_ = 5.5, P<0.05), caudate-putamen (F_2,11_ = 12.5, P<0.01) and thalamus (F_2,10_ = 4.7, P<0.05). Only in the hippocampus was there no significant age related change in relative binding. *Post hoc* Dunnett's t-tests indicated significant reductions (P<0.05) between PD2 and PD6 in the SCN and caudate-putamen, between PD6 and adult in the anterior hypothalamus, piriform cortex, caudate-putamen and thalamus, and between PD2 and adult in all regions. Levels of hybridization were highest in the piriform cortex and hippocampus at all ages. Comparing between age matched brain regions, the SCN, caudate-putamen and thalamus showed similar levels of hybridization, and with comparable age related declines in signals.

**Figure 4:**
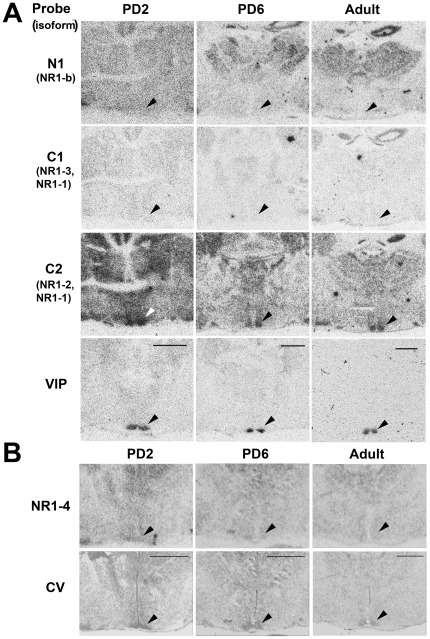
NR1 variable region gene expression in the Siberian hamster forebrain at different developmental ages. A. Autoradiographs of adjacent coronal sections from PD2 (left), PD6 (middle) and adult (right) hamster brain hybridized with ^35^S-labelled oligonucleotide probes N1, C1 and C2 complimentary to NR1 variable regions. Dense hybridization in the SCN only occurs with the C2 probe. Hybridization of the VIP probe identifies the position of the ventro-lateral SCN (bottom). Age-specific sections are from same representative animal. Arrows indicate position of SCN. Scale bars = 1000 µm. B. Hybridization of the NR1-4 probe (top), and adjacent cresyl violet stained section (bottom) in the hamster SCN. Autoradiographs of coronal sections taken through the forebrain at the level of the SCN (arrow) at the ages PD2 (left), PD6 (middle) and adult (right). Age-specific sections are from same representative animal. Cresyl violet Nissl stain (CV) identifies the SCN region by its dense regional staining. Scale bars = 1000 µm.

**Figure 5:**
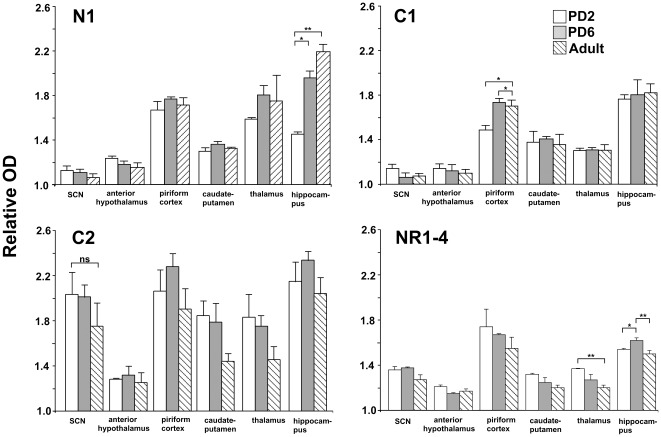
Quantification of NR1 variable region probe hybridization in forebrain regions in hamsters of ages PD2, PD6 and adult. Brain regions are suprachiasmatic nucleus (SCN), anterior hypothalamus, piriform cortex, caudate-putamen, thalamus, and hippocampus. Probes are N1, C1, C2 and NR1-4. Probe hybridization signal was measured as relative optical density (OD), representing relative levels of gene expression (OD of specific brain region divided by OD of corpus callosum in same section). Values represent the mean ± SEM relative OD values from 3 to 6 animals. *P<0.05 and **P<0.01 when regional relative OD is age compared animals (Dunnett's *post-hoc* t-test).

Figure 4A shows hybridization of the N1, C1 and C2 splice variant probes in the region of the SCN on PD2, PD6 and in the adult. NR1-4 splice variant probe hybridization is shown in Figure 4B. Hybridization of the N1 probe was relatively low/absent within the SCN at all ages. The piriform cortex, thalamus and hippocampus showed the greatest levels of hybridization of the N1 probe at all ages (Figure 4A and Figure 5), and an incremental elevation in expression was observed in the hippocampus during development, peaking in the adult (F_2,6_ = 85.6, P<0.001).

Hybridization of the C1 probe was also relatively low/absent within the SCN at all ages. The piriform cortex showed an elevation in expression after PD2 (F_2,6_ = 30.9, P<0.001). The relative binding in all other brain regions was conserved across ages. Regions showing greatest binding density were the piriform cortex and hippocampus (Figure 4A and Figure 5).

Hybridization of the C2 probe was relatively high within the SCN at all ages (Figure 4A and Figure 5). This conserved high level of hybridization was also observed in the piriform cortex and hippocampus. SCN binding levels remained similar throughout development (one-factor ANOVA: F_2,8_ = 0.84, n.s.). Relative grey scale values for C2 hybridization in striatum and thalamus did not decline significantly between neonatal and adult ages (one factor ANOVAs: caudate-putamen, F_2,8_ = 3.58, n.s.; thalamus, F_2,8_ = 2.30, n.s.).

Figure 4B shows hybridization of the NR1-4 splice variant probe in the region of the SCN on PD2, PD6 and in the adult. Low to moderate levels of hybridization of the NR1-4 probe occurred within the SCN at all ages (Figure 5). Regions showing greatest binding were the piriform cortex and hippocampus. Small but significant age-specific changes in expression were observed in the thalamus (F_2,6_ = 7.7, P<0.05) and hippocampus (F_2,6_ = 7.2, P<0.05). An incremental decline in expression occurred in the thalamus during development, whilst the hippocampus showed an elevated hybridization signal at PD6.

**Figure 6:**
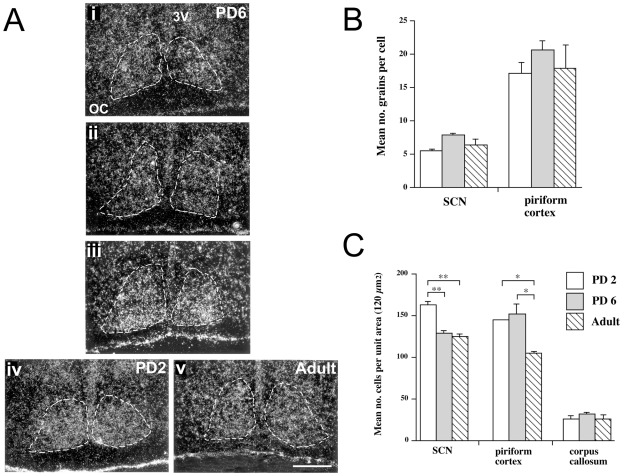
Assessment of NR1 probe hybridization by emulsion autoradiographs. (A) Dark-field photomicrographs from emulsion autoradiographs of C2 probe, depicting NR1_001_ (NR1-2a) receptor mRNA in PD2, PD6 and Adult Siberian hamster SCN. (i), (ii) and (iii) represent the rostral, medial and caudal SCN respectively at PD6. (iv) shows the medial SCN at PD2, and (v) shows the medial SCN in the adult. The region of the SCN is surrounded by dotted lines, and these were generated from describing the SCN region in corresponding Nissl-stained sections viewed under bright-field. OC, optic chiasm; 3 V, third ventricle. Scale bar = 200 µm. (B) Assessment of silver grain densities over SCN and piriform cortex cells on emulsion-dipped PAN probe hybridized sections. Mean ± SEM for number of grains per cell as measured in 120 µm^2^ area on brain sections from PD2 (white bars; n = 5), PD6 (grey bars; n = 5) and adult (hatched bars; n = 3) hamsters. (C) Assessment of cell density in the SCN, piriform cortex and corpus callosum on emulsion-dipped sections hybridized with PAN probe. Mean ± SEM for number of cells per 120 µm^2^ area on brain sections from PD2 (n = 5), PD6 (n = 5) and adult (n = 3) hamsters. *P<0.05 and **P<0.01 when cell density is age compared for groups (Dunnett's *post-hoc* t-test).

Examination of emulsion-dipped sections revealed hybridization of the PAN and C2 probes throughout the SCN as defined by cresyl violet staining of the same sections (Figure 6). Silver grain counts over SCN and piriform cortex cells and over the corpus callosum were taken from emulsion treated sections hybridized with the PAN probe. Age related differences in cellular grain density were analyzed by one-factor ANOVA (SCN, F_2,10_ = 3.48, n.s.; piriform cortex, F_2,10_ = 0.77, n.s.), which did not show a significant age-related change in the density of grains (Figure 6B). Levels of hybridization were 2–3 times higher in piriform cortex than in age-matched SCN, a relationship also noted in the analysis of the film autoradiographs (Figure 5). However, analysis of cell density in the SCN, piriform cortex and corpus callosum did reveal age-related differences (Figure 6C). One-factor ANOVA revealed a significant fall in the number of cells per unit area of SCN (F_2,10_ = 17.01, P<0.001) and piriform cortex (F_2,10_ = 4.97, P<0.05), but not in the corpus callosum (F_2,10_ = 0.40, n.s.). *Post hoc* t-tests indicated reductions between PD2 and PD6 in the SCN, between PD6 and adult in the piriform cortex, and between PD2 and adult in both the SCN and piriform cortex.

ISH controls were carried out to ascertain the specificity of hybridization by the C2 probe in the hamster, because of its high levels of binding within the SCN. Competition of the labeled C2 with 100 times excess cold probe completely blocked all binding in the SCN and all other brain forebrain regions. A labeled sense probe failed to bind to any regions, and prior treatment of labeled C2 hybridized sections with RNAse diminished binding (Figure S2).

To confirm the identity of the spice variants expressed in the SCN, we examined adult hamster SCN using oligonucleotide probes also designed to discriminate between splice variant isoforms but hybridizing to different regions of the NR1 rat gene sequence (Figure S3A). The relative hybridization signals of the NR1-b, NR1-1 and NR1-3 probes in the SCN were low, whilst that of the NR1-2 probe was moderate to high (Figure S3B, C). These data are consistent with the results of the N1/C1/C2/NR1-4 probe analysis, and confirms the absence within the SCN of isoforms containing the N region, the absence of isoforms containing both C1–C2 regions together, and the presence of isoforms containing the C2 region. These additional data firmly support that the major isoforms expressed in the adult Siberian hamster SCN are NR1_001_ (NR1-2a) and NR1_000_ (NR1-4a) (Table 1).

Immunohistochemical analysis revealed NR1-ir cell perikarya throughout the SCN and surrounding hypothalamus (Figure S4A). NR1-ir cells were distributed throughout the brain of the adult Siberian hamster, including cortex, hippocampus and cerebellum. Some of the cells showing striking immunoreactive perikarya were in the cortex, dentate gyrus and CA1-3 fields of the hippocampus and Purkinje cells of the cerebellum (Figure S4).

## Discussion

The expression of NR1 subunit in the adult rat SCN is well described [1], [14], [17], [26]. However, little has been documented in the Siberian hamster, a highly photoperiodic species and model organism of seasonality and puberty [28]. Consistent with the rat [15], [16], [47], [48], in the current study we observe the NR1 subunit within the hamster SCN. The PAN probe results revealed NR1 mRNA in the SCN of the adult rat and Siberian hamster. Our observations in the Siberian hamster also demonstrate that the NR1 gene is expressed in cells throughout the SCN, rather than being restricted to a particular zone and thus a specific neuronal phenotype. This is also consistent with studies in the rat [14], [49]. The developmental study using the PAN probe revealed the presence of NR1 mRNA throughout the postnatal period and, like the adult expression, it was noted in cells throughout the SCN rather than being restricted to a particular zone.

It cannot always be assumed that translation of mRNA invariably follows transcription. However, our immunohistochemical results revealed widespread distribution of NR1-ir cells in the SCN. This is consistent with other immunohistochemical studies in the Siberian hamster [46] and ligand binding studies in the rat using a radiolabeled non-competitive NMDA antagonist, MK801 [50]. In confirmation of the specificity of the NR1 antibody, the distribution of NR1-ir cells throughout the brain of the adult Siberian hamster corresponded with the original observations of Petralia *et al.* [45] that used the same antibody in the adult rat. Moreover, due to the specificity of the antisera for the C terminus of the NR1 subunit, it is likely that the immunohistochemical signal in the hamster SCN specifically reflects the expression of the NR1_001_ (NR1-2a) but not the NR1_000_ (NR1-4a) splice variant (see below).

The regional binding of the splice variant probes was similar between the Siberian hamster and rat, which suggests that the specificity of the probe is maintained between the two species. The results indicate the presence of the C2 (exon 22) cassette, but not the N1 (exon 5) or C1 (exon 21) cassettes in the SCN of both species. Thus, the predominant splice variants in the adult Siberian hamster and rat SCN would appear to be NR1_001_ (NR1-2a) and NR1_000_ (NR1-4a).

Examination of relative levels of hybridization of the PAN probe that identifies all NR1 mRNA variants, reveals an age-related decrease in NR1 expression in the SCN, anterior hypothalamus, caudate-putamen, thalamus and piriform cortex. It might then be expected that a decrease in the mRNA containing one or more of the splice-specific regions would underlie this observation and thus a developmental reduction in the binding of one or more of the region-specific probes would be apparent. However, apart from the reduction in NR1-4 probe (identifying isoforms NR1-4a and NR1-4b; Table 1) hybridization in the thalamus, this is not the case. Importantly, there is no significant decline in C2 (identifying isoforms NR1-2a, NR1-1a, NR1-2b and NR1-1b) or NR1-4 relative hybridization signals within the SCN at all ages.

The basis for the observed age-related changes in the relative grey scale values measured from autoradiographs could represent one or a combination of the following; altered cellular mRNA expression, altered density of cells expressing a particular mRNA transcript, different properties of the tissue that increase or reduce specific binding and non-specific binding independently irrespective of levels of the cellular probe specific mRNA. To address the first two possibilities together, cellular silver grain densities on photographic emulsion dipped sections were analyzed for hybridization of the PAN probe. This provided a measure of relative mRNA levels per cell rather than per brain region. Interestingly, the density of silver grains in the SCN and piriform cortex did not fall through development as might have been expected from the analysis of autoradiographic grey scale measures. However, a significant decrease between postnatal ages and the adult in the general cell density in the two brain regions was observed, presumably due to an expansion of the cell neuropil, i.e. areas that might not be expected to contain mRNA. Such a decrease in neuron density in the SCN during the neonatal period has been observed in the rat and Syrian hamster [30], [51]. This suggests that the age related decrease in autoradiographic grey scale measures for the SCN and piriform cortex reflect anatomical changes in the spacing of cells and not a pronounced change in the quantities of mRNA produced per cell. An analysis of hybridization signal in the corpus callosum was also undertaken to assess its application as a hybridization control region for the PAN probe. Silver grains were not associated with the low density of cells found in the white matter and thus they reflect non-specific background. The density of cells also did not differ between the three ages. Taking all these factors into consideration, it would appear that the age-related reduction in hybridization signal on the autoradiographs does not represent a significant reduction in mRNA levels in the SCN.

Laurie and Seeburg [25] and Monyer *et al.* [20] also describe developmental changes in binding levels of an NR1-pan probe in the rat brain using autoradiograph analysis. Their examination of brains from embryonic ages E14, E17 and E19, and postnatal ages PD2, PD8 and PD13 and adult, reveal a gradual increase in signal from low at E14 to peak at PD13, and in the adult this had declined. The authors explain the developmental drop in hybridization signal between PD13 and adult as a probable consequence of neuronal death and a reduction in expression by each neuron. The latter is supported by the statement that emulsion dipped slides showed that grain clusters over cells were much denser at PD13 than in the adult. Thus, this might explain the change in expression intensity in our study in other brain regions, such as the thalamus, but not for the SCN.

This decrease in hybridization signal was also demonstrated by Laurie and Seeburg [25] for all the NR1 isoform probes, detecting N1, C1 and C2 regions in different combinations; presence of N1 (identifying isoforms NR1-4b, NR-2b, NR1-3b and NR1-1b), absence of N1 (isoforms NR1-4a, NR1-2a, NR1-3a and NR1-1a), presence of C1 alone (isoforms NR1-3a and NR1-3b), presence of C2 alone (isoforms NR1-2a and NR1-2b), presence of C1 and C2 together (isoforms NR1-1a and NR1-1b), and the absence of C1 and C2 together (isoforms NR1-4a and NR1-4b) (Table 1). All probes were reported to show hybridization signals that rose until PD13 and had fallen in the adult. Whilst none of these probes apart from NR1-4 share identical sequences to the probes used in the current investigation on development, the N1 probe, detecting for the presence of the N1 region, is similar to the probe used by Seeburg and Laurie [25]. In the current investigation the only such significant reductions in hybridization intensity were in the thalamus and hippocampus with the NR1-4 probe.

Our data in the hamster SCN reveals a high level of relative hybridization signal for the C2 probe and a relatively lower signal for the NR1-4 probe. Whilst it would be tempting to suggest that the ratio expression of NR1_001_>NR1_000_, it is accepted that it is difficult to directly compare the levels of binding of different probes and correlate this to mRNA concentrations. This is because, in addition to differences in the abundance of the target mRNA, differences can occur in the specific activity of the probes, in the relative preservation of the particular mRNA in the histological processing and in the efficiency of the probe binding to the target [52], [53]. However, in support of the notion that NR1_001_ is highly expressed in the hamster SCN are the results from using alternative probes in adult tissue including one that specifically identified the C2 region coupled with the absence of the C1 region (NR1-2 probe). Similar to the DELL-II probe, NR1-2 hybridization signal in the SCN region was strong, and higher than the NR1-4 probe.

The presence of C2 and NR1-4, but not N1 or C1 probe hybridization within the SCN throughout the postnatal period, strongly suggests that the dominance of the NR1_001_ (NR1-2a) and NR1_000_ (NR1-4a) isoforms is conserved throughout postnatal and adult life. The conserved nature of the regional expression of an isoform, as demonstrated by the SCN, is consistent with observations of other forebrain regions in the rat, such as the basal ganglia. This suggests that whilst there may be developmental changes in relative levels of the hybridization signal, there is little change in their regional distributions. Moreover, these patterns appear to be established around birth [25].

It is interesting to note that the NR1_001_ isoform would render the receptor subunit relatively insensitive to phosphorylation by protein kinase C [24], [54]. This is in contrast to the splice variants common in the hippocampus that contain the N1 and C1 cassettes, which have been shown capable of extensive phosphorylation, a modification that might contribute to the process of long-term potentiation [55]. It might be predicted that the stability of the response to synaptic glutamatergic activation from cycle to cycle would be an important feature of the process of resetting a circadian clock. The NR1_X00_ (NR1-4a and NR1-4b) isoforms and NR2B subunit have been shown to be colocalized with neuronal nitric oxide synthase (nNOS), and shown to be physically coupled with nNOS [56]–[58]. As nitric oxide (NO) signaling is involved in photic entrainment of the circadian system this is particularly relevant [59], [60]. The NR1_X00_ isoform possesses the shortest C-terminal tail, is not retained in the endoplasmic reticulum and is translocated directly to the cell membrane surface [61], [62]. As the NR1_X00_ subunit in the rat is rhythmically expressed, it has been suggested that this might facilitate the transfer of NR2B subunit and nNOS to the cell surface and contribute to the night photosensitivity of cell membranes in the SCN [26].

These data are generally consistent with the study of NR1 splice variants in a recent study of adult and developing rat SCN [26]. In the rat SCN, NR1_001_ (probes NR1a, NR1-2) and NR1_000_ (probes NR1a, NR1-4) are ubiquitously expressed throughout postnatal stages and in the adult. However, in contrast to the Siberian hamster SCN, Bendová *et al.* [26] additionally report the presence of the NR1_011_ isoform (probes NR1a and NR1-1) in the rat that persists at constant levels throughout development and in the adult, and report developmental changes in the NR1_010_ (probes NR1a and NR1-3) and NR1-E isoforms, specifically their elevation during embryonic and postnatal stages but low/absent expression in the adult. As the C1 cassette possesses regulatory and signaling elements, its absence in the Siberian hamster and its presence in the rat indicates that there may be functional differences in the glutamatergic signaling in the SCN between the two species.

Whilst the role of the NMDAR including NR1 is accepted as important in the regulation of retino-recipient development in the vertebrate optic tectum and mammalian visual thalamus and superior colliculus [5], [7], [63]–[66], it is not apparent whether changes in the NR1 isoform are involved in these processes. A change in the relative expression of NR1 isoforms during SCN development would afford different functional properties to the NMDA receptor complex that could potentially contribute to this process. In the current study we do not observe such changes in splice variant expression during early postnatal period when the RHT begins to innervate the SCN and eventually undergo pruning of terminals [10], [11]. However, a recent examination of the NR1 gene in embryonic brain, has revealed a novel truncated N-terminal form, the NR1-E variant [67]. Whilst not examined in our analysis in the Siberian hamster, the developmental expression of this isoform in the rat SCN [26], in which its expression peaks at E20, is present during the postnatal period, but is absent in the adult, suggests a specific role during SCN maturation including neuronal innervation and synaptogenesis.

The NMDA receptor can also contribute to non-glutamatergic pathways involved in the modulation of photic entrainment, such as those utilizing pituitary adenylyl cyclase activating peptide (PACAP), neuropeptide Y (NPY) and 5-hydroxytryptophan (5-HT) [68]. In the case of PACAP, activation of PACAP receptors results in increased cyclic adenosine monophosphate (cAMP) and this then can activate NMDARs through phosphorylation on NR1 Ser897 [69]. However, as Ser897 is localized in the C1 cassette, which is absent or in low expression in the Siberian hamster SCN, but present in the NR1_011_ and NR1_010_ isoforms in the rat [26], this suggests a key difference in PACAP signaling between the species.

It is perhaps not surprising that the NR1 isoforms are conserved throughout postnatal life to adult if it is important in the regulation of the circadian clock by light. The early onset of a cellular response to light in the Siberian hamster SCN suggests that the circadian clock can be light entrained early in the postnatal period [10]. If this is the case, then a mature NMDA receptor system might allow similar responses in the developing animal as it would in the mature adult. Even the presence of NR1 in the SCN at PD2 may be of no surprise given that the SCN is to be responsive to photic cues during the night of PD3 [10]. Interestingly, native glutamate and glutamate agonists in combination are able to induce c-Fos and phosphorylation of cAMP response element-binding protein (CREB) in SCN cell culture and SCN tissue slices, prepared from PD1-2 Syrian hamsters [70], [71]. This would suggest that the glutamatergic receptor systems in the SCN are well developed early on in life, several days before the RHT begins to innervate the SCN [11], [32] and before a cellular response to light can occur *in vivo* [10], [72]. Therefore, the early presence of glutamate receptors within the SCN relates to their capacity of being activated by glutamate leading to the production of robust cellular events. This reveals that the major limitation to the development of photic phase-shifting capabilities is a sufficient radiation of the retinal afferents within the SCN, recruiting an appropriate population of responsive SCN neurons [10], [11].

The biological significance of a response to photic cues for a burrowing rodent is questionable in the first few days of life, but Siberian hamster pups do become ambulatory prior to opening of the eye lids and may therefore become exposed to photic cues in the environment. Certainly by PD14 Siberian hamster pups are capable of independent survival, and neuroanatomical development of the SCN and its stimulation by photic signals suggest that hamsters are capable of responding to the environmental photoperiod by this age [9]–[11]. One possibility is that the significance of the early acquisition of response to ambient photoperiod relates to the highly seasonal nature of Siberian hamsters [28], [73]. The circadian system underlies the generation of a pattern of melatonin secretion from the pineal gland, which then provides a neurochemical measure of night length. In several species of rodent including the Siberian hamster, it is clear that the pattern of melatonin during postnatal development is measured relative to that received from the maternal pineal gland while *in utero*, thereby indicating the direction of change of photoperiod [29], [74], [75]. This provides an accurate temporal signal so that reproductive, metabolic and growth responses, which are optimal for the season, are engaged accordingly. Thus, it might be expected that rodents would evolve the capacity to respond and entrain to environmental photoperiod at an early age such that accurate regulation of the pineal gland is accomplished independently of maternal photoperiodic time influences. Furthermore, it is becoming apparent that photoperiodic and pharmacological programming of the developing SCN can result in permanent effects upon the adult circadian system and related regulation of metabolism, posing important considerations for human health [76]–[78].

In conclusion, these data reveal that the predominant splice variants expressed within the Siberian hamster SCN are NR1_001_ (NR1-2a) and NR1_000_ (NR1-4a), and that this pattern of gene expression is conserved throughout postnatal development. Therefore we conclude that a switch in NR1 isoform does not underlie or is not produced by the dramatic changes in that occur within the developing SCN. This consistency in the presence of the NR1 isoforms highlights the importance of having a stable response to synaptic glutamatergic activation. As the SCN contains the master circadian pacemaker that is reset by light, and is critical in photoperiodic time measurement, this would allow for a stable response to photic signals from the environment that are intrinsically variable over the 24 hr day and calendar year.

## Supporting Information

Figure S1 **Hybridization of NR1 variable region probes in rat forebrain.** Shown are the PAN (top), N1, C1 and C2 (bottom) probes in the adult rat brain. Autoradiograph of alternate coronal sections taken through the forebrain at the level of the SCN from a representative animal. a, anterior hypothalamus; cp, caudate putamen; p, piriform cortex; s, suprachiasmatic nucleus; t, thalamus. Scale bar = 1000 µm.
(PDF)

Figure S2 **Control studies for** ***in situ*** **hybridization of C2 probe in PD6 Siberian hamster.** A, B, labeled C2 probe; C, D, labeled C2 probe with excess cold C2 probe; E, F, RNAse treatment and labeled C2 probe; G, H, labeled C2 sense probe. Autoradiographs of alternate coronal sections taken through the forebrain at the level of the SCN from a representative animal. Whole section (left) and higher magnification of the hypothalamus including SCN (right). Arrow indicates position of SCN. Scale bars = 500 µm.
(PDF)

Figure S3 **Confirmation of the identity of the NR1 splice variants expressed in the adult Siberian hamster SCN.** (A) Oligonucleotide probes were used designed to discriminate between splice variant isoforms but hybridizing to alternative regions of the NR1 rat gene sequence. NR1 mRNA structure with the position of three alternatively spliced exons in the mRNA (upper) and the complementary positions of the additional splice-specific oligonucleotide probes (lower) (see Figure 1). The NR1-b probe identifies the presence of the N region. Whilst specific to a different DNA region, NR1-b is equivalent to the N1 probe (Figure 1). NR1-1 probe detects for the simultaneous presence of both C1 and C2 regions, whilst NR1-2 detects the simultaneous presence of the C1 and absence of the C2 region. Conversely the NR1-3 probe detects the simultaneous absence of the C1 and presence of the C2 region. (B) Representative hybridization of the NR1-b, NR1-1, NR1-2 and NR1-3 probes in adult hamster in the region of SCN and anterior hypothalamus (top) and in forebrain (bottom). SCN was identified from corresponding adjacent cresyl violet stained sections. Autoradiographs of coronal sections taken through the forebrain at the level of the SCN (arrow) and anterior hypothalamus. Note that hybridization signals for the NR1-b, NR1-1 and NR1-3 probes were detected in other forebrain regions. c, cerebral cortex; cp, caudate-putamen; h, hippocampus; p, piriform cortex; t, thalamus; OC, optic chiasm; 3 V, third ventricle. Scale bars = 200 µm (top) and 1000 µm (bottom). (C) Quantification of hybridization in adult hamster SCN with NR1 probes NR1-b, NR1-1, NR1-2 and NR1-3. Probe hybridization signal was measured as relative optical density (OD), representing relative levels of gene expression (OD of specific brain region divided by OD of corpus callosum in same section). Values represent the mean ± SD relative OD values from 2 animals.
(PDF)

Figure S4 **NR1-immunoreactive cells in the adult Siberian hamster brain.** Coronal sections through A, the SCN and anterior hypothalamus; B, caudate-putamen; C, cerebral cortex; D, piriform cortex; E, cerebellum (arrow indicates a Purkinje cell); and F, hippocampus. DG, dentate gyrus; OC, optic chiasm; 3 V, third ventricle. Scale bar for A to E = 100 µm, scale bar for F = 200 µm.
(PDF)

Table S1 **Oligonucleotide probes used in the Siberian hamster and rat.**
(PDF)

## References

[1] Mikkelsen JD, Larsen PJ, Mick G, Vrang N, Ebling FJP (1995). Gating of retinal inputs through the suprachiasmatic nucleus: role of excitatory neurotransmission.. Neurochem Int.

[2] Morin LP (1994). The circadian visual system.. Brain Res Reviews.

[3] Ebling FJP (1996). The role of glutamate in the photic regulation of the suprachiasmatic nucleus.. Prog Neurobiol.

[4] Constantine-Paton M, Cline HT, Debski E (1990). Patterned activity, synaptic convergence, and the NMDA receptor in the developing visual pathways.. Annu Rev Neurosci.

[5] Hofer M, Constantine-Paton M (1994). Regulation of N-methyl-D-aspartate (NMDA) receptor function during the rearrangement of developing neuronal connections.. Prog Brain Res.

[6] Moriya T, Horikawa K, Akiyama M, Shibata S (2000). Correlative association between N-methyl-D-aspartate receptor-mediated expression of period genes in the suprachiasmatic nucleus and phase shifts in behavior with photic entrainment of clock in hamsters.. Mol Pharmacol.

[7] Rauschecker JP (1991). Mechanisms of visual plasticity - Hebb synapses, NMDA receptors, and beyond.. Physiol Rev.

[8] Kleinschmidt A, Bear MF, Singer W (1987). Blockade of “NMDA” receptors disrupts experience-dependent plasticity of kitten striate cortex.. Science.

[9] Duffield GE, Ebling FJP (1998). Maternal entrainment of developing circadian system in the Siberian hamster (Phodopus sungorus).. J Biol Rhythms.

[10] Duffield GE, Dickerson JM, Alexander IHM, Ebling FJP (1995). Ontogeny of a photic response in the suprachiasmatic nucleus in the Siberian hamster (Phodopus sungorus).. Eur J Neurosci.

[11] Duffield GE, McNulty S, Ebling FJP (1999). Anatomical and functional characterization of a dopaminergic system in the suprachiasmatic nucleus of the neonatal Siberian hamster.. J Comp Neurol.

[12] Weaver DR, Reppert SM (1995). Definition of the developmental transition from dopaminergic to photic regulation of c-fos gene expression in the rat suprachiasmatic nucleus.. Mol Brain Res.

[13] Grosse J, Davis FC (1999). Transient entrainment of a circadian pacemaker during development by dopaminergic activation in Syrian hamsters.. Brain Res Bull.

[14] Mikkelsen JD, Larsen PJ, Ebling FJP (1993). Distribution of N-methyl D-aspartate (NMDA) receptor mRNAs in the rat suprachiasmatic nucleus.. Brain Res.

[15] van den Pol AN, Hermans-Borgmeyer I, Hofer M, Ghosh P, Heinemann S (1994). Ionotropic glutamate-receptor gene expression in hypothalamus: localization of AMPA, kainate and NMDA receptor RNA with in situ hybridization.. J Comp Neurol.

[16] Gannon RL, Rea MA (1994). In situ hybridization of antisense mRNA oligonucleotides for AMPA, NMDA and metabotropic glutamate receptor subtypes in the rat suprachiasmatic nucleus at different phases of the circadian cycle.. Mol Brain Res.

[17] Watanabe M, Inoue Y, Sakimura K, Mishina M (1993). Distinct distributions of five NMDA receptor channel subunit mRNAs in the forebrain.. J Comp Neurol.

[18] Ghosh PK, Baskaran N, van den Pol AN (1997). Developmentally regulated gene expression of all eight metabotropic glutamate receptors in hypothalamic suprachiasmatic and arcuate nuclei-a PCR analysis.. Dev Brain Res.

[19] Watanabe M, Inoue Y, Sakimura K, Mishina M (1992). Developmental changes in distribution of NMDA receptor channel subunit mRNAs.. NeuroReport.

[20] Monyer H, Burnashev N, Laurie DJ, Sakmann B, Seeburg PH (1994). Developmental and regional expression in the rat brain and functional properties of four NMDA receptors.. Neuron.

[21] van den Pol AN, Kogelman L, Ghosh P, Liljelund P, Blackstone C (1994). Developmental regulation of the hypothalamic metabotropic glutamate receptor mGluR1.. J Neurosci.

[22] Cull-Candy S, Brickley S, Farrant M (2001). NMDA receptor subunits: diversity, development and disease.. Curr Opin Neurobiol.

[23] Durand GM, Bennett MVL, Zukin RS (1993). Splice variants of the N-methyl-D-aspartate receptor NR1 identify domains involved in regulation by polyamines and protein kinase C.. Proc Natl Acad Sci USA.

[24] Tingley WG, Roche KW, Thompson AK, Huganir RL (1993). Regulation of NMDA receptor phosphorylation by alternative splicing of the C-terminal domain.. Nature.

[25] Laurie DJ, Seeburg PH (1994). Regional and developmental heterogeneity in splicing of the rat brain NMDAR1 mRNA.. J Neurosci.

[26] Bendová Z, Sumová A, Mikkelsen JD (2009). Circadian and developmental regulation of N-methyl-d-aspartate-receptor 1 mRNA splice variants and N-methyl-d-aspartate-receptor 3 subunit expression within the rat suprachiasmatic nucleus.. Neuroscience.

[27] Manta G, Spathis AD, Taraviras S, Kouvelas ED, Mitsacos A (2011). Age and Visual Experience-dependent Expression of NMDAR1 Splice Variants in Rat Retina.. Neurochem Res.

[28] Ebling FJP (2010). Photoperiodic regulation of puberty in seasonal species.. Mol Cell Endocrinol.

[29] Weaver DR, Reppert SM, Reppert SM (1989). Maternal communication of daylength information to the fetus.. Research in perinatal medicine IX: development of circadian rhythmicity and photoperiodism in mammals.

[30] Moore RY, Shibata S, Bernstein ME, Reppert SM (1989). Developmental anatomy of the circadian system.. Research in perinatal medicine IX: development of circadian rhythmicity and photoperiodism in mammals.

[31] Moore RY, Bernstein ME (1989). Synaptogenesis in the rat suprachiasmatic nucleus demonstrated by electron-microscopy and synapsin-1 immunoreactivity.. J Neurosci.

[32] Speh JC, Moore RY (1993). Retinohypothalamic tract development in the hamster and rat.. Dev Brain Res.

[33] Duffield GE, Hastings MH, Ebling FJP (1998). Investigation into the regulation of the circadian system by dopamine and melatonin in the Siberian hamster (Phodopus sungorus).. J Neuroendocrinol.

[34] Ohta H, Honma S, Abe H, Honma K (2002). Effects of nursing mothers on rPer1 and rPer2 circadian expressions in the neonatal rat suprachiasmatic nuclei vary with developmental stage.. Eur J Neurosci.

[35] Ohta H, Honma S, Abe H, Honma K (2003). Periodic absence of nursing mothers phase-shifts circadian rhythms of clock genes in the suprachiasmatic nucleus of rat pups.. Eur J Neurosci.

[36] El-Hennamy R, Mateju K, Bendova Z, Sosniyenko S, Sumova A (2008). Maternal control of the fetal and neonatal rat suprachiasmatic nucleus.. J Biol Rhythms.

[37] Sumová A, Bendová Z, Sládek M, El-Hennamy R, Laurinová K (2006). Setting the biological time in central and peripheral clocks during ontogenesis.. FEBS Letters.

[38] Matejů K, Bendová Z, El-Hennamy R, Sládek M, Sosniyenko S (2009). Development of the light sensitivity of the clock genes Period1 and Period2, and immediate-early gene c-fos within the rat suprachiasmatic nucleus.. Eur J Neurosci.

[39] Hastings MH, Duffield GE, Maywood ES, Smith EJD, Ebling FJP (1998). Entrainment of the circadian system of mammals by non-photic cues.. Chronobiol Int.

[40] Monyer H, Sprengel R, Schoepfer R, Herb A, Higuchi M (1992). Heteromeric NMDA receptors: molecular and functional distinction of subtypes.. Science.

[41] Standaert DG, Testa CM, Young AB, Penney JB (1994). Organization of N-methyl-D-aspartate glutamate receptor gene expression in the basal ganglia of the rat.. J Comp Neurol.

[42] Ivell R, Richter D (1984). Structure and comparison of the oxytocin and vasopressin genes from rat.. Proc Natl Acad Sci USA.

[43] Nishizawa M, Hayakawa Y, Yanaihara N, Okamoto H (1985). Nucleotide-sequence divergence and functional constraint in VIP precursor messenger-RNA evolution between human and rat.. FEBS Letters.

[44] Jensen AM, Chiu SY (1993). Expression of glutamate receptor genes in white matter: developing and adult rat optic nerve.. J Neurosci.

[45] Petralia RS, Yokotani N, Wenthold RJ (1994). Light and electron microscope distribution of the NMDA receptor subunit NMDAR1 in the rat nervous system using a selective anti-peptide antibody.. J Neurosci.

[46] Ebling FJP, Alexander IHM, Urbanski HF, Hastings MH (1995). Effects of N-methyl-D-aspartate (NMDA) on seasonal cycles of reproduction, body weight and pelage colour in the male Siberian hamster.. J Neuroendocrinol.

[47] Kus L, Handa RJ, Sanderson JJ, Kerr JE, Beitz AJ (1995). Distribution of NMDAR1 receptor subunit mRNA and [125I]MK801 binding in the hypothalamus of intact, castrate and castrate-DHTP treated male rats.. Mol Brain Res.

[48] Sato K, Mick G, Kiyama H, Tohyamas M (1995). Expression patterns of a glutamate-binding protein in the rat central nervous system: comparison with N-methyl-D-asparatate receptor subunit 1 in rat.. Neuroscience.

[49] Ishida N, Matsui M, Mitsui Y, Mishina M (1994). Circadian expression of NMDA receptor mRNAs, e3 and z1 in the suprachiasmatic nucleus of rat brain.. Neurosci Lett.

[50] Hartgraves MD, Fuchs JL (1994). NMDA receptor binding in rodent suprachiasmatic nucleus.. Brain Res.

[51] Müller C, Torrealba F (1998). Postnatal development of neuron number and connections in the suprachiasmatic nucleus of the hamster.. Dev Brain Res.

[52] Young WS, Kuhar MJ, Uhl GR (1986). Quantitative in situ hybridization and determination of mRNA content.. In situ hybridization in brain.

[53] Smolen AJ, Beaston-Wimmer P, Chesselet MF (1990). Quantitative analysis of in situ hybridization using image analysis.. In situ hybridization histochemistry.

[54] Zukin RS, Bennett MVL (1995). Alternatively spliced isoforms of the NMDAR1 receptor subunit.. Trends Neurosci.

[55] Thomas KL, Davis S, Laroche S, Hunt SP (1994). Regulation of the expression of NR1 NMDA glutamate receptor subunits during hippocampal LTP.. Neuroreport.

[56] Kornau HC, Schenker LT, Kennedy MB, Seeburg PH (1995). Domain interaction between NMDA receptor subunits and the postsynaptic density protein PSD-95.. Science.

[57] Brenman JE, Chao DS, Gee SH, McGee AW, Craven SE (1996). Interaction of nitric oxide synthase with the postsynaptic density protein PSD-95 and alpha1-syntrophin mediated by PDZ domains.. Cell.

[58] Weiss SW, Albers DS, Iadarola MJ, Dawson TM, Dawson VL (1998). NMDAR1 glutamate receptor subunit isoforms in neostriatal, neocortical, and hippocampal nitric oxide synthase neurons.. J Neurosci.

[59] Ding JM, Chen D, Weber ET, Faiman LE, Rea MA (1994). Resetting the biological clock: mediation of nocturnal circadian shifts by glutamate and NO.. Science.

[60] Golombek DA, Agostino PV, Plano SA, Ferreyra GA (2004). Signaling in the mammalian circadian clock: the NO/cGMP pathway.. Neurochem Int.

[61] Okabe S, Miwa A, Okado H (1999). Alternative splicing of the C-terminal domain regulates cell surface expression of the NMDA receptor NR1 subunit.. J Neurosci.

[62] Horak M, Wenthold RJ (2009). Different roles of C-terminal cassettes in the trafficking of full-length NR1 subunits to the cell surface.. J Biol Chem.

[63] Colonnese MT, Constantine-Paton M (2006). Developmental period for N-methyl-D-aspartate (NMDA) receptor-dependent synapse elimination correlated with visuotopic map refinement.. J Comp Neurol.

[64] Zhao JP, Phillips MA, Constantine-Paton M (2006). Long-term potentiation in the juvenile superior colliculus requires simultaneous activation of NMDA receptors and L-type Ca2+ channels and reflects addition of newly functional synapses.. J Neurosci.

[65] Miskevich F, Doench JG, Townsend MT, Sharp PA, Constantine-Paton M (2006). RNA interference of Xenopus NMDAR NR1 in vitro and in vivo.. J Neurosci Methods.

[66] Cramer KS, Sur M (1996). The role of NMDA receptors and nitric oxide in retinogeniculate development.. Prog Brain Res.

[67] Campusano JM, Andrés ME, Magendzo K, Abarca J, Tapia-Arancibia L (2005). Novel alternative splicing predicts a truncated isoform of the NMDA receptor subunit 1 (NR1) in embryonic rat brain.. Neurochem Res.

[68] Colwell CS, Michel S, Itri J, Rodriguez W, Tam J (2004). Selective deficits in the circadian light response in mice lacking PACAP.. Am J Physiol Regul Integr Comp Physiol.

[69] Llansola M, Sánchez-Pérez AM, Montoliu C, Felipo V (2004). Modulation of NMDA receptor function by cyclic AMP in cerebellar neurones in culture.. J Neurochem.

[70] McNulty S, Schurov IL, Sloper PJ, Hastings MH (1998). Stimuli which entrain the circadian clock of the neonatal Syrian hamster in vivo regulate the phosphorylation of the transcription factor CREB in the suprachiasmatic nucleus in vitro.. Eur J Neurosci.

[71] Schurov IL, McNulty S, Best JD, Sloper PJ, Hastings MH (1999). Glutamatergic induction of CREB phosphorylation and Fos expression in primary cultures of the suprachiasmatic hypothalamus in vitro is mediated by co-ordinate activity of NMDA and non-NMDA receptors.. J Neuroendocrinol.

[72] Kaufman CM, Menaker M (1994). Ontogeny of light-induced Fos-like immunoreactivity in the hamster suprachiasmatic nucleus.. Brain Res.

[73] Ebling FJP (1994). Photoperiodic differences during development in the dwarf hamsters Phodopus sungorus and Phodopus campbelli.. Gen Comp Endocrinol.

[74] Horton TH, Stachecki SA, Stetson MH (1990). Maternal transfer of photoperiodic information in Siberian hamsters. IV: Peripubertal reproductive development in the absence of maternal photoperiodic signals during gestation.. Biol Reprod.

[75] Lee TM, Spears N, Tuthill CR, Zucker I (1989). Maternal melatonin treatment influences rates of neonatal development of meadow vole pups.. Biol Reprod.

[76] Ciarleglio CM, Axley JC, Strauss BR, Gamble KL, McMahon DG (2011). Perinatal photoperiod imprints the circadian clock.. Nat Neurosci.

[77] Kennaway DJ (2002). Programming of the fetal suprachiasmatic nucleus and subsequent adult rhythmicity.. Trends Endocrinol Metab.

[78] Varcoe TJ, Wight N, Voultsios A, Salkeld MD, Kennaway DJ (2011). Chronic phase shifts of the photoperiod throughout pregnancy programs glucose intolerance and insulin resistance in the rat.. PLoS One.

[79] Kováčiková Z, Sládek M, Bendová Z, Illnerová H, Sumová A (2006). Expression of Clock and Clock-Driven Genes in the Rat Suprachiasmatic Nucleus during Late Fetal and Early Postnatal Development.. J Biol Rhythms.

[80] Hollmann M, Boulter J, Maron C, Beasley L, Sullivan J (1993). Zinc potentiates agonist-lnduced currents at certain splice variants of the NMDA receptor.. Neuron.

[81] Sugihara H, Moriyoshi K, Ishii T, Masu M, Nakanishi S (1992). Structures and properties of seven isoforms of the NMDA receptor generated by alternative splicing.. Biochem Biophys Res Commun.

[82] Nakanishi N, Axel R, Shneider NA (1992). Alternative splicing generates functionally distinct N-methyl-D-aspartate receptors.. Proc Natl Acad Sci USA.

