# Supplementary figures and images for: Conserved Expression of the Glutamate NMDA Receptor 1 Subunit Splice Variants during the Development of the Siberian Hamster Suprachiasmatic Nucleus
Source: PLoS One. 2012 May 31;7(5):e37496. doi: 10.1371/journal.pone.0037496 (PMC3365105; doi:10.1371/journal.pone.0037496)

# rat

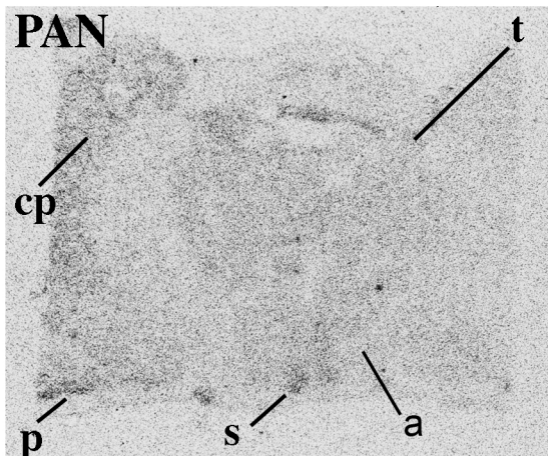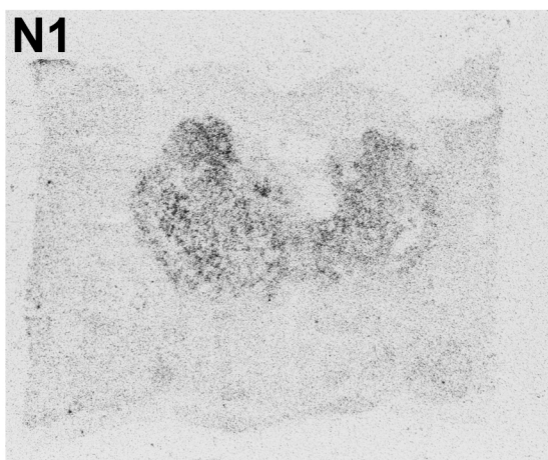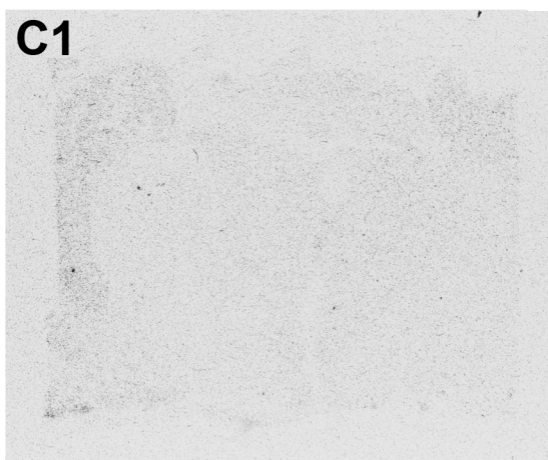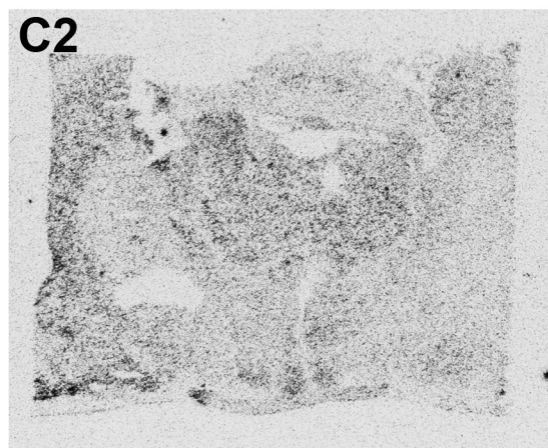

Supplement: Figure S1 — Hybridization of NR1 variable region probes in rat forebrain. Shown are the PAN (top), N1, C1 and C2 (bottom) probes in the adult rat brain. Autoradiograph of alternate coronal sections taken through the forebrain at the level of the SCN from a representative animal. a, anterior hypothalamus; cp, caudate putamen; p, piriform cortex; s, suprachiasmatic nucleus; t, thalamus. Scale bar = 1000 µm. (PDF) [file pone.0037496.s001.pdf]

A

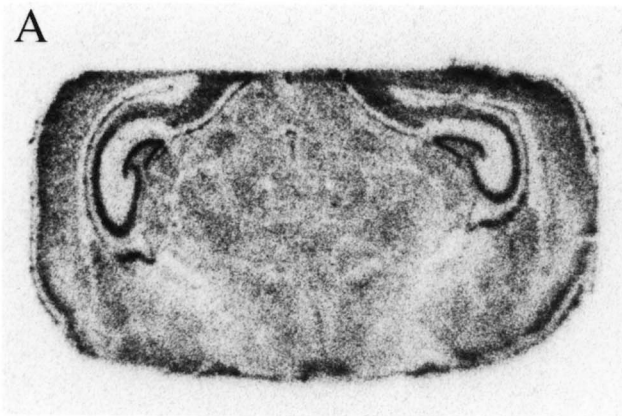

B

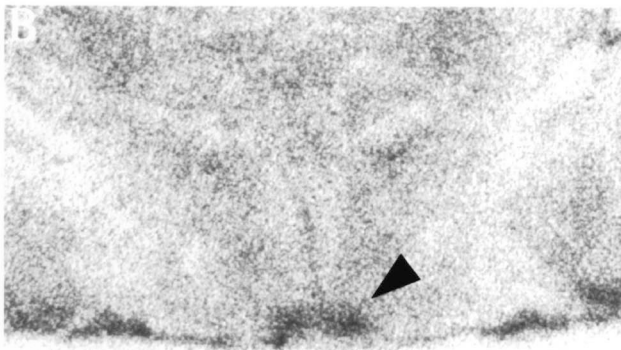

C

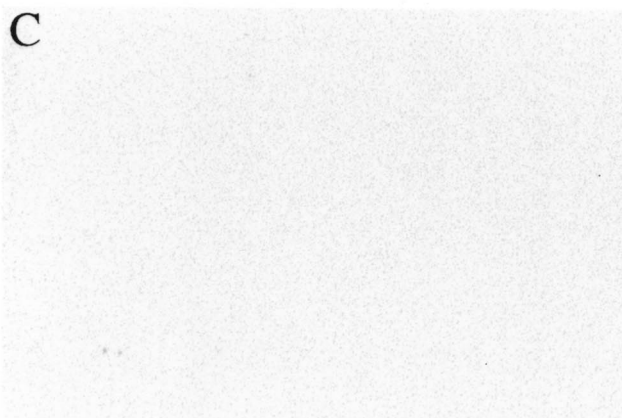

D

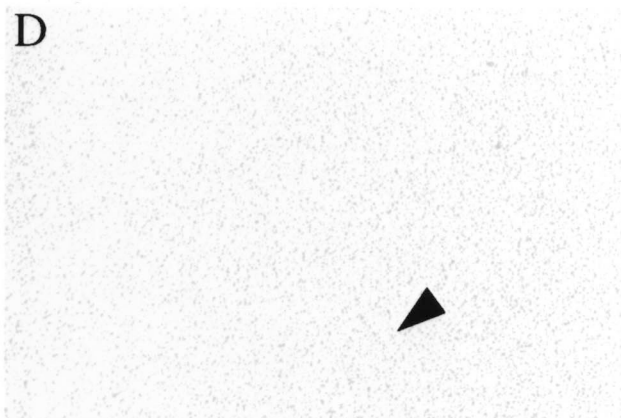

E

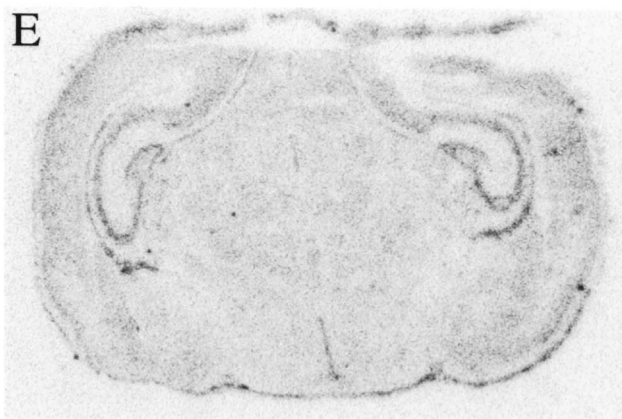

F

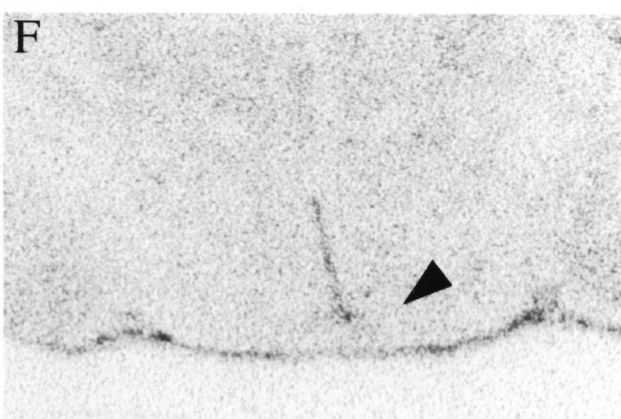

G

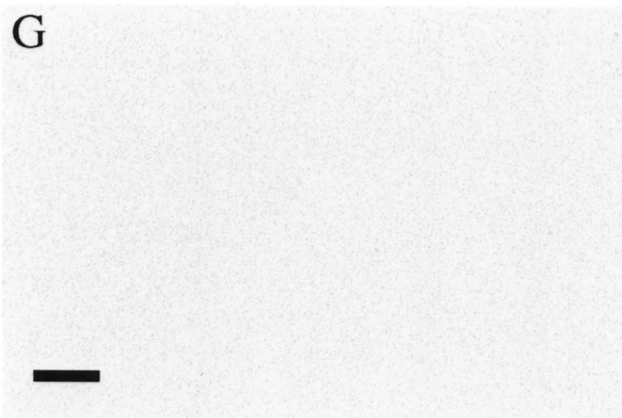

H

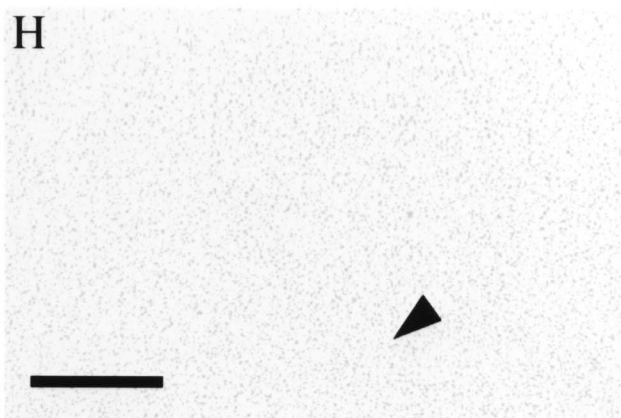

Supplement: Figure S2 — Control studies for in situ hybridization of C2 probe in PD6 Siberian hamster. A, B, labeled C2 probe; C, D, labeled C2 probe with excess cold C2 probe; E, F, RNAse treatment and labeled C2 probe; G, H, labeled C2 sense probe. Autoradiographs of alternate coronal sections taken through the forebrain at the level of the SCN from a representative animal. Whole section (left) and higher magnification of the hypothalamus including SCN (right). Arrow indicates position of SCN. Scale bars = 500 µm. (PDF) [file pone.0037496.s002.pdf]

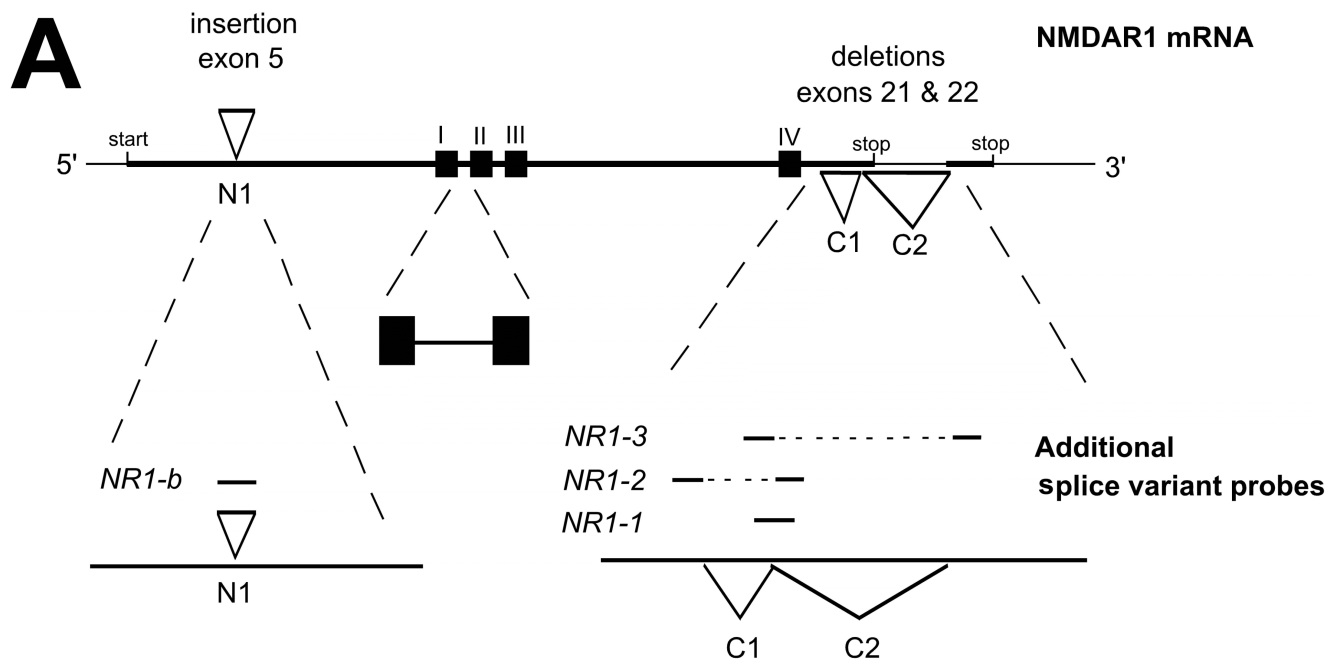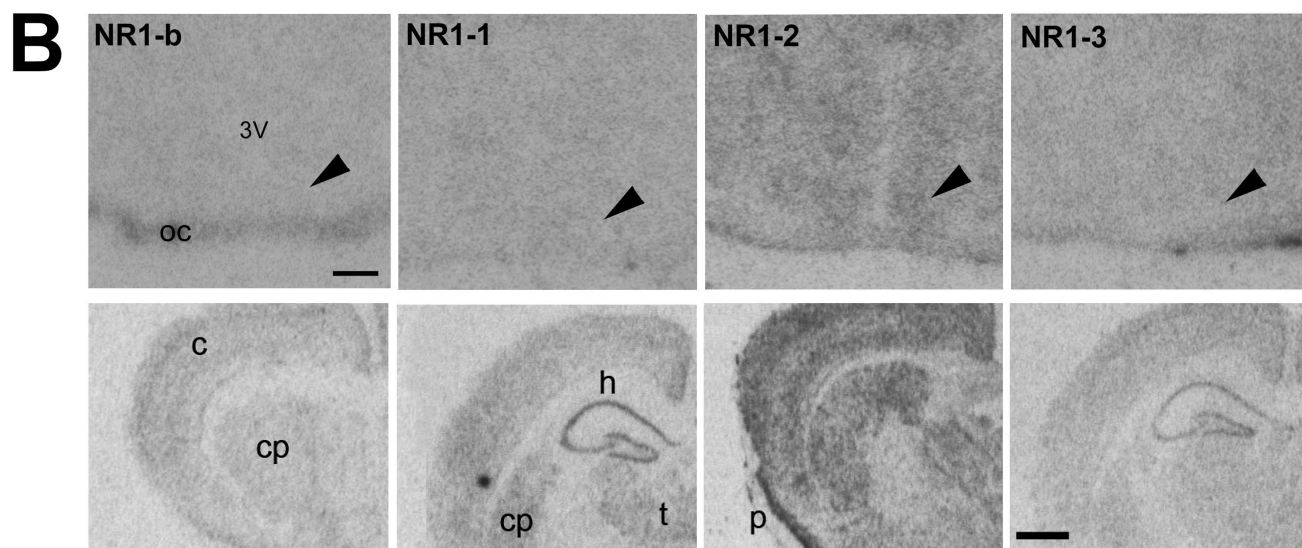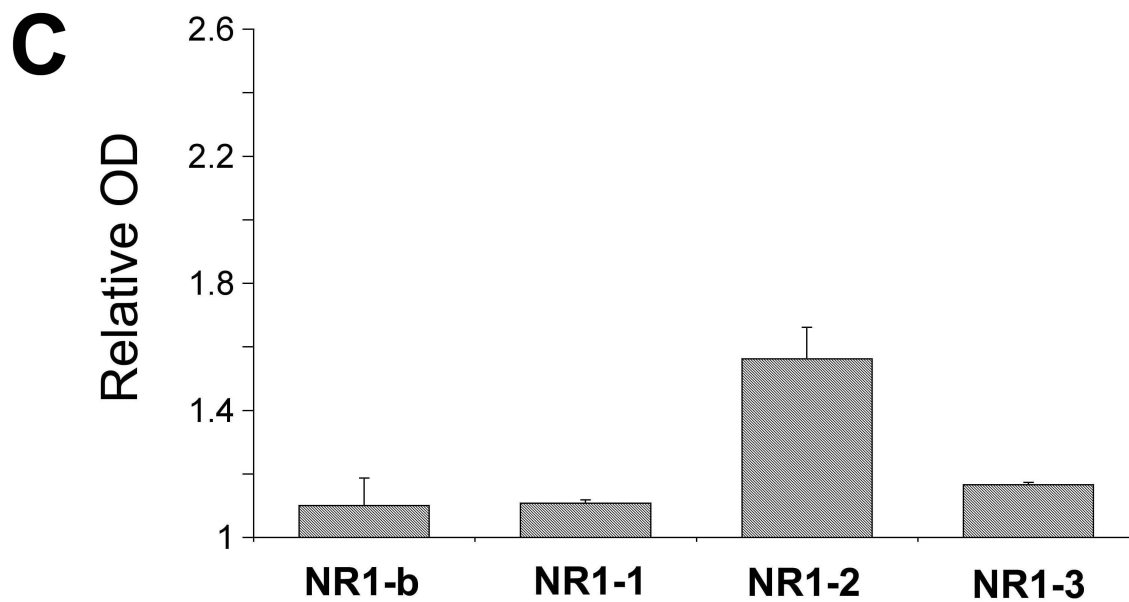

Supplement: Figure S3 — Confirmation of the identity of the NR1 splice variants expressed in the adult Siberian hamster SCN. (A) Oligonucleotide probes were used designed to discriminate between splice variant isoforms but hybridizing to alternative regions of the NR1 rat gene sequence. NR1 mRNA structure with the position of three alternatively spliced exons in the mRNA (upper) and the complementary positions of the additional splice-specific oligonucleotide probes (lower) (see Figure 1). The NR1-b probe identifies the presence of the N region. Whilst specific to a different DNA region, NR1-b is equivalent to the N1 probe (Figure 1). NR1-1 probe detects for the simultaneous presence of both C1 and C2 regions, whilst NR1-2 detects the simultaneous presence of the C1 and absence of the C2 region. Conversely the NR1-3 probe detects the simultaneous absence of the C1 and presence of the C2 region. (B) Representative hybridization of the NR1-b, NR1-1, NR1-2 and NR1-3 probes in adult hamster in the region of SCN and anterior hypothalamus (top) and in forebrain (bottom). SCN was identified from corresponding adjacent cresyl violet stained sections. Autoradiographs of coronal sections taken through the forebrain at the level of the SCN (arrow) and anterior hypothalamus. Note that hybridization signals for the NR1-b, NR1-1 and NR1-3 probes were detected in other forebrain regions. c, cerebral cortex; cp, caudate-putamen; h, hippocampus; p, piriform cortex; t, thalamus; OC, optic chiasm; 3 V, third ventricle. Scale bars = 200 µm (top) and 1000 µm (bottom). (C) Quantification of hybridization in adult hamster SCN with NR1 probes NR1-b, NR1-1, NR1-2 and NR1-3. Probe hybridization signal was measured as relative optical density (OD), representing relative levels of gene expression (OD of specific brain region divided by OD of corpus callosum in same section). Values represent the mean ± SD relative OD values from 2 animals. (PDF) [file pone.0037496.s003.pdf]

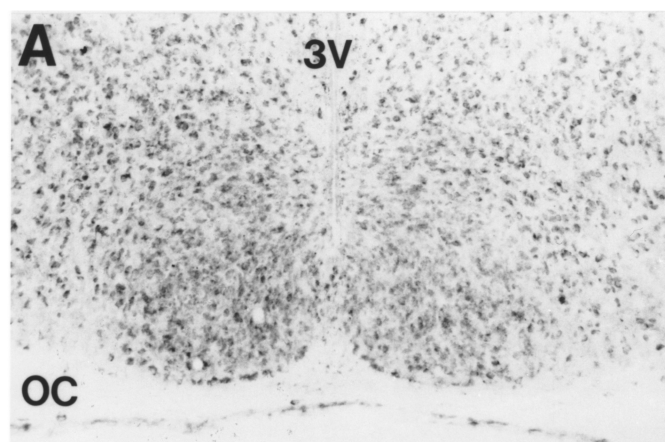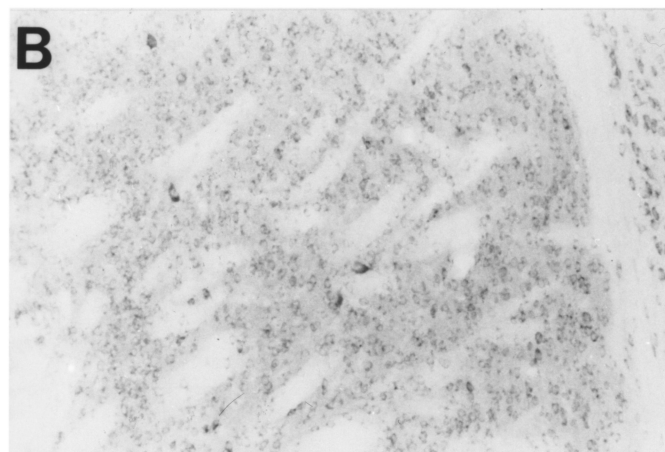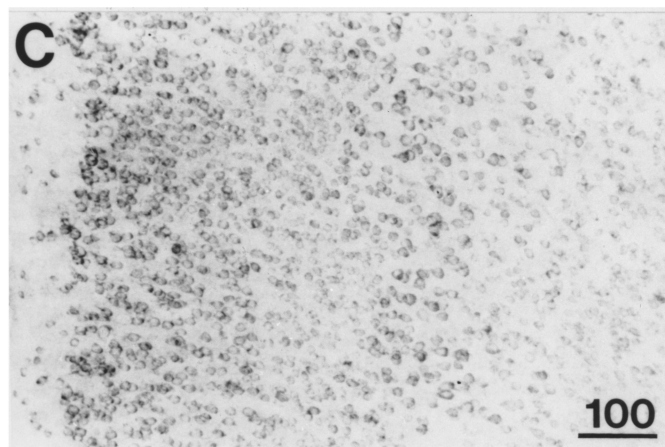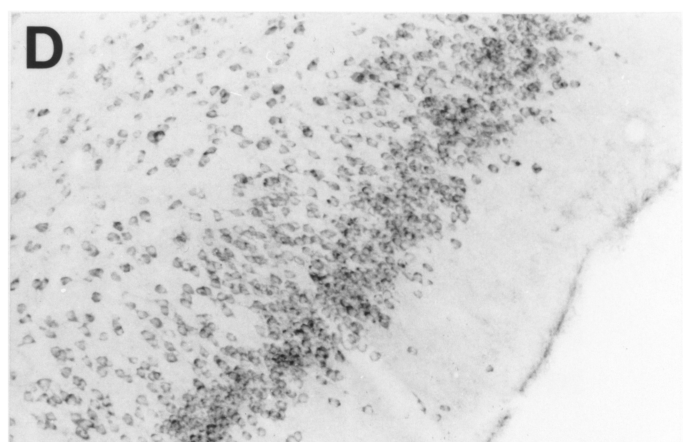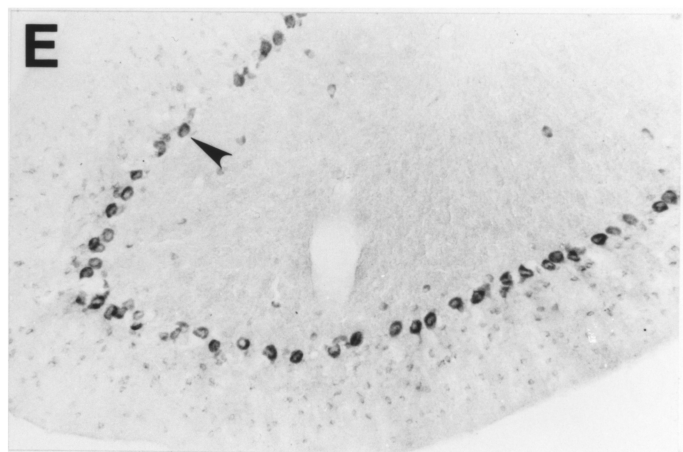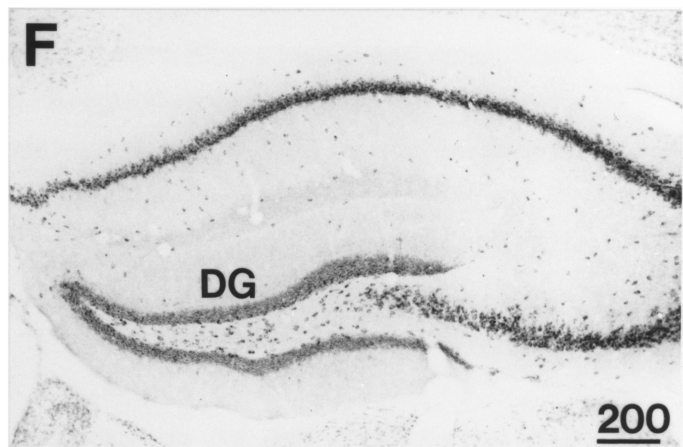

Supplement: Figure S4 — NR1-immunoreactive cells in the adult Siberian hamster brain. Coronal sections through A, the SCN and anterior hypothalamus; B, caudate-putamen; C, cerebral cortex; D, piriform cortex; E, cerebellum (arrow indicates a Purkinje cell); and F, hippocampus. DG, dentate gyrus; OC, optic chiasm; 3 V, third ventricle. Scale bar for A to E = 100 µm, scale bar for F = 200 µm. (PDF) [file pone.0037496.s004.pdf]
