# Supplementary material for: Conserved Expression of the Glutamate NMDA Receptor 1 Subunit Splice Variants during the Development of the Siberian Hamster Suprachiasmatic Nucleus
Source: PLoS One. 2012 May 31;7(5):e37496. doi: 10.1371/journal.pone.0037496 (PMC3365105; doi:10.1371/journal.pone.0037496)
Supplement: Table S1 — Oligonucleotide probes used in the Siberian hamster and rat. (PDF) [file pone.0037496.s005.pdf]

Oligonucleotide	Ref	5' 3'
NR1 PAN		CTCCTCCTCACTGTTCACCTTGAATCGGCCAAAGGGACTGAAGCGGTC
NR1 N1 (presence of N region)	[41]	CTTGGGTCCGCGCTTGTTGTCATAGGACAGTTGGTCGAGGTTTTC
NR1 C1 (presence of C1 region)	[41]	CGTGTCTTTGGAGGACCTACGTCTCTTGAAGCTGGAGGCCAGGGT
NR1 C2 (presence of C2 region)	[41]	TATGACGGGAACACAGCTGCAGCTGGCCCTCCTCCCTCTCAATAG
NR1 C2 sense	[41]	CTATTGAGAGGGAGGAGGGCCAGCTGCAGCTGTGTTCCC GTCATA
NR1-4 (absence C1 & C2 regions)	[25]	GATATCAGTGGGATGGTACTGCTGCAGGTTCTTCCTCCAC
NR1-b (presence of N region)	[25]	GCGCTTGTTGTCATAGGACAGTTGGTCGAGGTTTTCATAG
NR1-1 (presence C1 & C2 regions)	[25]	TCCACCCCCGGTGCTCGTGTCTTTGGAGGACCTACGTCTC
NR1-2 (C1 absent, C2 present)	[25]	TCCACCCCCGGTGCTCTGCAGGTTCTTCCTCCACACGTT
NR1-3 (C1 present, C2 absent)	[25]	GATATCAGTGGGATGGTACTGCGTGTCTTTGGAGGACCTA
AVP		GTAGACCCGGGGCTTGGCAGAATCCACGGACTCTTGTGTCCCAGCCAG
VIP		GTGTCGTTTGACCGGCACGGGGTCTTCCGAGATGCTACTGCTGAT

Table S1. **Oligonucleotide probes used in the Siberian hamster and rat.** The PAN probe was designed to recognize all NR1 mRNAs and to hybridize to the rat mRNA sequence encoding the region between the transmembrane domains II and III and part of domain II [80]. Splice variant probe sequences derived from rat were as described in Standaert *et al*. [41] and Laurie and Seeburg [25]. Probes were designed to detect AVP and VIP from rat mRNA sequences [42, 43].
